# Universal Bulk-Fill Composites: An Investigation into the Efficiency of Rapid Curing with Reversible Addition–Fragmentation-Chain Transfer (RAFT)-Mediated Polymerisation

**DOI:** 10.3390/ma18194489

**Published:** 2025-09-26

**Authors:** Nicoleta Ilie

**Affiliations:** Department of Conservative Dentistry, University Hospital, Ludwig-Maximilians-University, Goethestr. 70, D-80336 Munich, Germany; nicoleta.ilie@med.uni-muenchen.de

**Keywords:** degree of conversion, dental composites, RAFT polymerization

## Abstract

Novel universal bulk-fill composites with reversible addition–fragmentation chain-transfer (RAFT)-modulated polymerization continue the trend towards increasing simplification of the restoration process to facilitate the reconstruction of deep posterior restorations in 4 mm increments as well as anterior restorations through improved aesthetics. This study aims to assess the suitability of such materials for rapid curing (3 s) with high-radiant emittance in terms of degree of conversion (DC) and polymerization kinetics at relevant depths (2 mm vs. 4 mm). For this purpose, two newly introduced bulk-fill universal composites (Tetric^®^ plus Flow and Tetric^®^ plus Fill) were compared with already established fast-curing composites (Tetric^®^ Power Flow and Tetric^®^ Power Fill). DC was measured in real time over 300 s using ATR-FTIR spectroscopy. The temporal DC evolution was modelled using an exponential sum function. Novel bulk-fill composites showed DC results that were independent of the measured sample depth or curing mode. The polymerization kinetics of all composites are somewhat slower in the gel phase at moderate irradiance or when measured at deeper layers, but compensate for the differences in the glass phase, finally reaching equivalent DC values by the end of the 300-s observation period. These novel composites are therefore suitable for rapid curing (3 s) at high irradiance.

## 1. Introduction

The trend in the development of restorative materials, especially dental polymer-based composites, is clearly moving toward treatment simplification. Since treatment time is expensive and it is unlikely that material costs can be significantly reduced, the logical consequence was to reduce the number of treatment steps and consequently the treatment time. Compromises in quality and aesthetics are non-negotiable in modern dentistry. Therefore, considerable efforts are being invested in the development of modern technologies and tailor-made solutions for novel, improved dental materials with an increasingly broader range of indications.

One of the first movements that became noticeable in this regard, which is not only accepted but has already been clinically proven in both primary [1,2,3] and permanent molar restorations [2,4,5], was the application of composites in thick increments of up to 4–5 mm, e.g., in bulk. Aesthetic limitations caused by the increased translucency of the bulk-fill materials required for better deep curing are gradually being eliminated. The size of the fillers—which was greatly increased in the first bulk-fill materials to reduce the polymer-filler interface and thus the scattering of light, to allow more light to penetrate deeper—has progressively been reduced to improve aesthetics and polishability. As modern bulk-fill composites become increasingly more aesthetic, a new trend has recently emerged towards truly universal composites that enable both rapid filling of deep posterior restorations in thick layers and sufficient aesthetic for use in the anterior region.

In addition to the facts described above, efforts have also been made to shorten the light exposure duration. This goal has been pursued for decades and has already achieved considerable success, as the polymerization time of most composites on the market has been reduced to only 10–20 s. Efforts to further shorten exposure duration failed decades ago because short curing times of 1–3 s were far insufficient to ensure adequate polymerization in the depth of a restoration [6,7]. Apart from changing the photoinitiators to Norrish Type I photoinitiators (e.g., monoacylphosphine oxide Lucirin-TPO), which require new curing equipment as they need violet light for initiation (wavelength range 395–415 nm) [8], the only feasible step at that moment was to change the polymerization mechanism, i.e., to implement the reversible addition–fragmentation chain-transfer (RAFT)-mediated polymerization [9,10] instead of the conventional free radical polymerization. Indeed, ultrafast curing (3 s) at high irradiance in composites with RAFT-mediated polymerization proved to be sufficient [11,12] and equivalent to the conventional 10 s irradiation at moderate irradiance [13], while extending the curing time to 20 s did not result in any measurable improvements in quasi-static and viscoelastic parameters, cell toxicity [14], or degree of cure [13]. Furthermore, the mechanical parameters and the degree of conversion up to a depth of 4 mm of such composites were found to be equivalent to composites cured by conventional radical polymerization [10], even after ageing. High-intensity curing raises concerns about temperature rise that can lead to pulp injury [15]. However, rapid curing (3 s) was shown to result in the smallest temperature rise and the shortest time to maximum temperature compared to moderate-intensity 10 s and 20 s curing modes [13]. Furthermore, high-intensity curing in 3 s has been shown to improve bond strength in RAFT-mediated composites, including ageing [16]. The effect of rapid curing in 3 s at high irradiance is expressed in a higher initial shrinkage rate, but a lower (Tetric Power Flow) or equal (Tetric PowerFill) linear shrinkage 15 min after polymerization [12]. Furthermore, it was shown that the rapid curing of Tetric Power Flow in 3 s at high irradiance has an adverse effect on the marginal integrity in dentin but not in enamel, while Tetric PowerFill behaves similarly regardless of the curing conditions [17].

Although the implementation of RAFT-mediated polymerization [18] initially appeared to be a simple and logical consequence of transferring innovative technological achievements from other industries [19] to dental materials, significant difficulties arose, leading to a delay of at least 15 years. The main problem was that RAFT-mediated polymerization requires a special RAFT agent, usually a thiocarbonylthio compound [20], which imparts either colour or odour [19] to dental materials, which is unacceptable for their clinical application. A suitable agent without the mentioned side effects needed to be synthesised and is still today not commercially available [20]. This fact has led to this novel polymerization method being implemented in only a few materials, particularly in materials from two dental companies [21]. Nevertheless, RAFT-mediated polymerization offers a number of advantages [18] for dental materials, as all components of an existing composite can be retained, including the monomers, additives, or photoinitiator systems. In addition, the existing light curing units as well as the adhesive systems can continue to be used and there are no differences in application compared to the known and routine methods, which do not require any adjustments or changes for the practitioner.

Recently, bulk-fill materials with RAFT-mediated polymerization and improved aesthetics have been introduced. These materials are referred to as universal composites, making them suitable not only for posterior restorations, as was previously the case, but also for anterior restorations. In addition, they are said to be capable of ultrafast curing (in 3 s) at high irradiances, thus shortening the restoration process. Therefore, the aim of this study is to verify whether ultrafast polymerization of these materials in only 3 s at high irradiance is comparable in terms of degree of conversion to more conventional polymerization in 10 s at moderate irradiance. The null hypothesis therefore states that the ultrafast curing (3 s) at high radiant emittance has no influence on a) the degree of conversion measured 300 s post-polymerization or b) the polymerization kinetic of the novel bulk-fill composites compared to a usual 10 s polymerization at moderate irradiance. Furthermore, it is assumed that c) these universal composites behave similarly to their predecessors, already established composites, with regard to the above-mentioned parameters.

## 2. Materials and Methods

### 2.1. Materials

Four micro hybrid, light-curing, bulk-fill, polymer-based dental composite materials (composites) were selected that implemented RAFT polymerization mechanisms. Two of them are regular composites and the other two are flowable composites. In addition, Tetric PowerFill and Tetric Power Flow are already established materials and Tetric Plus and Tetric Plus Flow have been recently released. Details on the composition and material characteristics are presented in Table 1.

### 2.2. Methods

The employed light curing unit (LCU) Bluephase PowerCure (Ivoclar Vivadent, Schaan, Lichtenstein) was characterised spectrophotometrically to determine the emission spectrum, incident irradiance, and radiation exposure. The degree of conversion was determined in real time for 300 s using ATR-FTIR spectroscopy, at 2 mm and 4 mm depths.

#### 2.2.1. Spectroscopy: LCU Characteristics

The LCU was characterised using a laboratory spectrophotometer (MARC; acronym of Managing Accurate Resin Curing System, Blue light Analytics Inc., Halifax, NS, Canada), referenced by the National Institute of Standards and Technology (NIST). The spectrometer uses a 3648-element linear charge-coupled device (CCD) array detector with high-speed electronics (Ocean optic, Largo, FL, USA) and was calibrated with a NIST-traceable light source (300−1050 nm) from Ocean Optics. The system uses a CC3-UV Cosine Corrector (Ocean optic, Largo, FL, USA) to collect radiation over a 180° field of view, thus mitigating the effects of optical interference associated with light collection sampling geometry.

Irradiance was determined three times by placing the LCU centrally and perpendicularly to the 3.9 mm diameter spectrophotometer sensor. The irradiance measured by directly placing the LCU on the sensor represents the incident irradiance, i.e., the irradiance that hits the sample surface during curing. The sensor was triggered at 20 mW. Irradiances in the wavelength range 360–540 nm were recorded individually at a rate of 16 frames/s to separate the violet and blue wavelength ranges. The exposure time was either 3 s in the 3sCure mode or 10 s in the High-Power mode. The radiant exposure for the individual exposure modes and wavelength ranges was calculated from the integral of the irradiance over the exposure time.

#### 2.2.2. ATR-FTIR Spectroscopy: Degree of Conversion (DC)

DC was measured using Attenuated Total Reflection-Fourier Transform Infrared (ATR-FTIR) spectroscopy (Nicolet iS50, Thermo Fisher, Madison, WI, USA). The recording was carried out in real time, with a spectrum being recorded every 0.4 s over the entire 300 s at the bottom of 2 and 4 mm high samples (n = 6) [10]. Teflon moulds (3 mm diameter, 2 and 4 mm deep) were filled with the composite material to be tested. For this purpose, the unpolymerized composite was applied directly onto the diamond ATR crystal in the respective mould and covered with a transparent matrix strip. The samples were cured either for 3 s in the 3sCure mode or for 10 s in the High Power curing mode.

Then the variation in the peak height ratio of the absorbance intensities of the methacrylate carbon–carbon double bond peak (C=C) at 1637 cm^−1^ relative to the aromatic C=C double bond peak at 1608 cm^−1^, which serves as an internal standard that remains constant, was monitored during the exposure and post-polymerization phase and used to calculate DC according to the following Equation:

(1) $${\mathrm{DC}}_{\mathrm{Peak}}\%\;=\;[1\;-\;\frac{{\left(1637\;cm^{-1}/1608\;cm^{-1}\right)}_{\mathrm{Peak}\;\mathrm{height}\;\mathrm{after}\;\mathrm{curing}}}{{\left(1637\;cm^{-1}/1608\;cm^{-1}\right)}_{\mathrm{Peak}\;\mathrm{height}\;\mathrm{before}\;\mathrm{curing}}}]\times 100$$

The increase in DC over time, from the beginning of light exposure to 300 s post-polymerisation, was then mathematically fitted using the sum of two exponential functions:

(2) $$y=a*(1-e^{-bx})+c*(1-e^{-dx})$$

where the parameters *a*, *b*, *c*, *d* are modulation factors of the exponential sum function.

### 2.3. Statistical Analyses

The normal distribution of the data was confirmed by the Shapiro-Wilk test. A parametric approach was used for further analysis. DC was compared using single-factor and multifactorial analysis of variance (ANOVA) and Tukey’s post hoc test for significant differences (HSD), with an alpha risk of 5%. A multivariate analysis (general linear model) assessed the effect of the parameter material, depth, and curing mode as well as their interaction products on DC. For significant independent factors, the partial eta-squared values (η_P_^2^) indicate the effect strength on the measured data; the higher the η_P_^2^, the stronger the effect (SPSS Inc. Version 29.0, Chicago, IL, USA).

## 3. Results

### 3.1. Light Curing Unit (LCU) Characteristics

**Figure 1:**
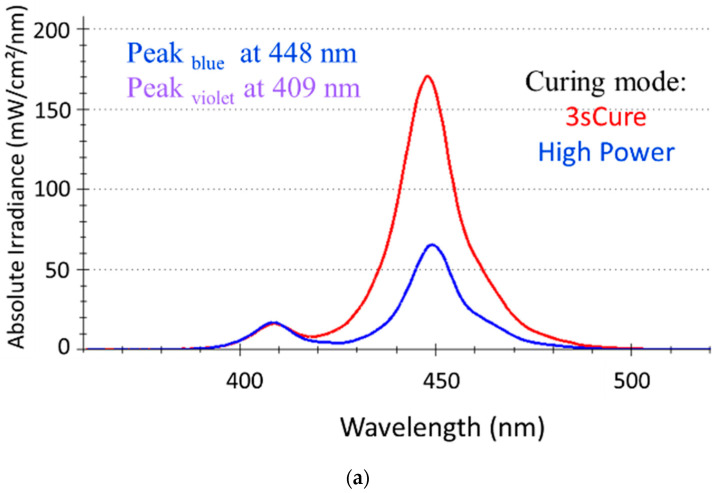

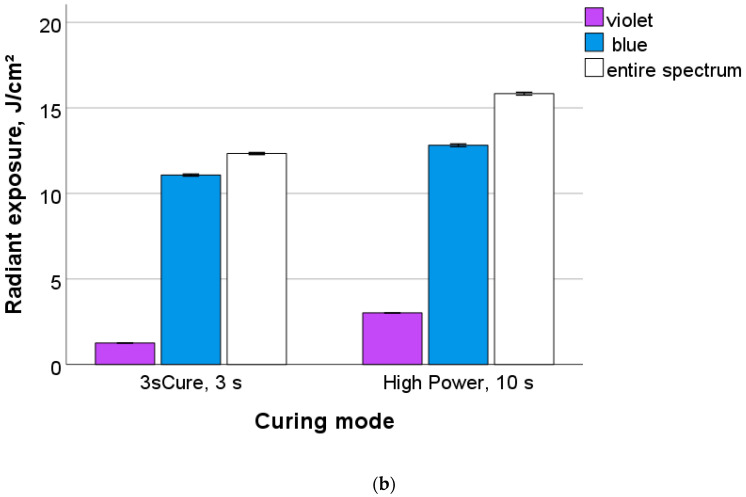
LCU characteristics as a function of the used curing mode: (**a**) spectral distribution for the curing modes 3sCure (3 s exposure duration) and High Power (10 s exposure duration); (**b**) Radiant exposure (for the entire spectrum, the blue and violet wavelength ranges) for the used curing modes.

The LCU used for polymerization is a violet–blue LED LCU with a peak in the blue wavelength range at 448 nm and one in the violet wavelength range at 409 nm (Figure 1a). The emission spectrum and irradiance of the LCU in the violet wavelength range are perfectly overlapping for both tested curing modes (3sCure and High Power). In the blue wavelength range, the emission spectra are similar, while the irradiance is about three times higher in the 3sCure mode (Figure 1b). Maximal irradiance, which corresponds to placing the LCU centrally and directly above the sensor (exposure distance = 0 mm), was (4132.5 ± 16.69) mW/cm^2^ for the 3sCure mode and (1585.9 ± 12.8) mW/cm^2^ for the High Power mode. This value also represents the incident irradiance to the surface of the composite specimens. The corresponding total radiant exposure at the 3 s exposure duration in the 3sCure mode was (12.3 ± 0.03) J/cm^2^, summing up the radiant exposure in the violet wavelength range (1.2 ± 0.01) J/cm^2^ and in the blue wavelength range (11.1 ± 0.03) J/cm^2^. The analogous parameters for the 10 s exposure in the High Power mode were as follows: total radiant exposure (15.8 ± 0.03) J/cm^2^, radiant exposure (violet) (3.0 ± 0.01) J/cm^2^, and radiant exposure (blue) (12.8 ± 0.03) J/cm^2^.

### 3.2. Degree of Conversion (DC) and Kinetic of Polymerisation

**Figure 2:**
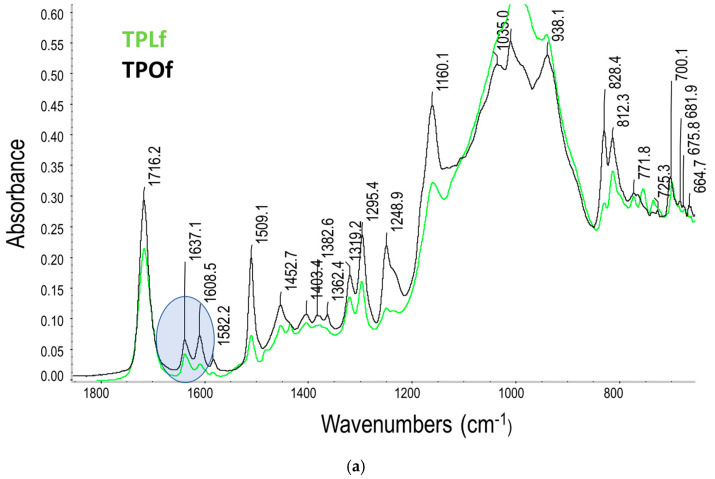

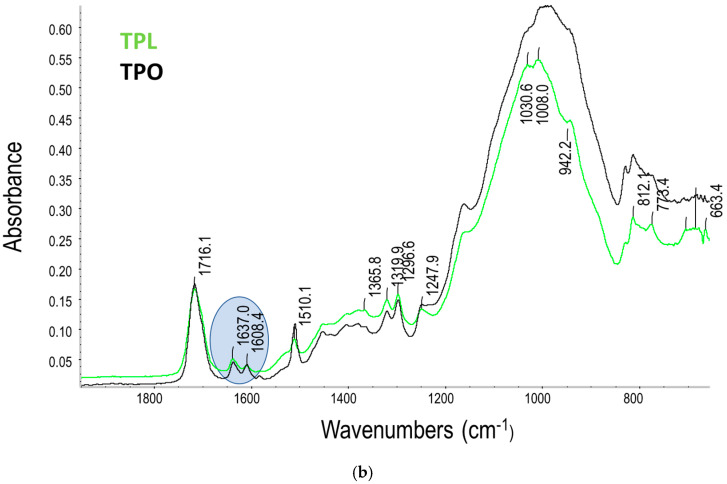
FTIR spectra of the analysed materials: (**a**) flowable composites TPLf and TPOf, and (**b**) regular composites TPL and TPO (see abbreviation in Table 1).

The degree of conversion was expressed as the conversion of the aliphatic carbon–carbon double bond peak (C=C) from the unpolymerized composite paste during the measured 300 s. The interaction of the chemical bonds with the infrared beam causes specific vibrations, such as stretching and bending, which result in characteristic absorption bands. The FTIR spectra of the four analysed materials in an unpolymerized state are summarised in Figure 2a,b, with the characteristic peaks used for DC evaluation marked by a blue ellipse.

**Figure 3:**
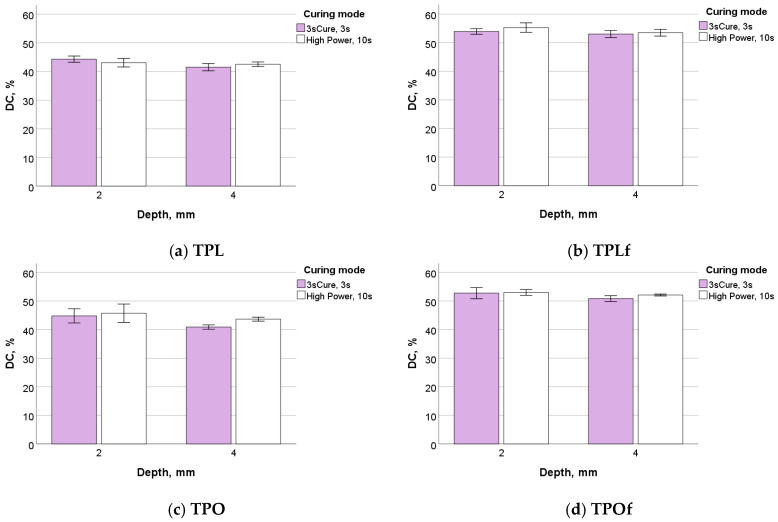
Degree of conversion (%) as a function of material, depth, and curing mode.

The DC at 300 s post-polymerisation was statistically assessed in a multifactorial analysis. A significant (*p* < 0.001) and strong influence of the material on the DC was observed (high partial eta-squared value, η_P_^2^ = 0.935), while the effect of depth and curing mode was very low (η_P_^2^ = 0.097). None of the binary or ternary parameter combinations have a significant effect (*p* > 0.05) on DC. The DC results are presented for all examined materials, depths, and curing modes in detail below (Figure 3).

**Figure 4:**
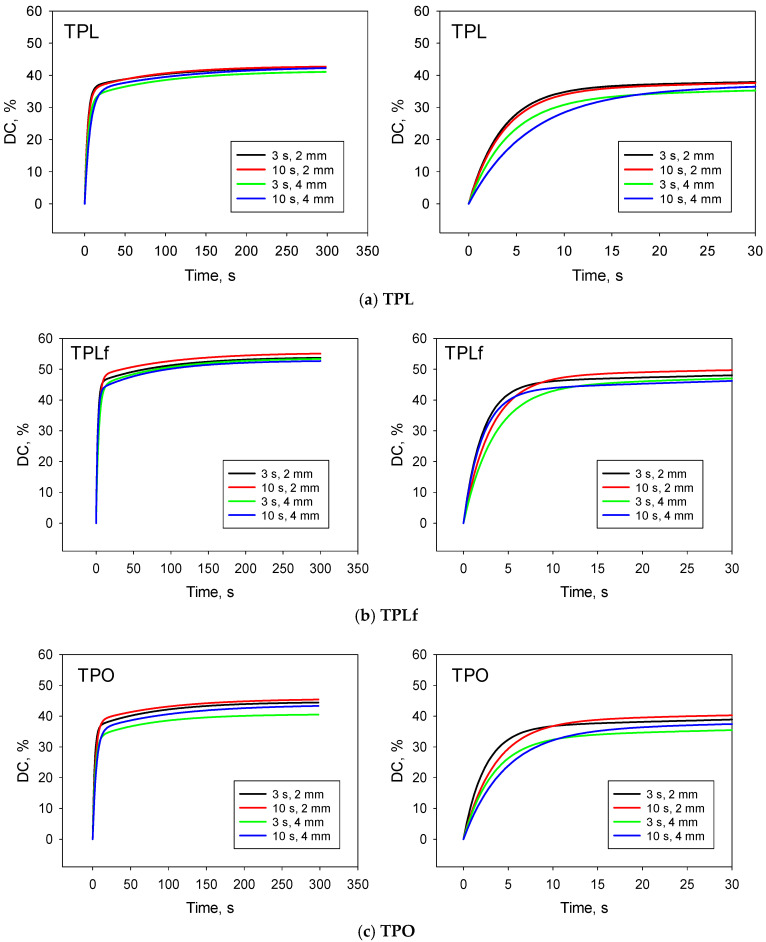

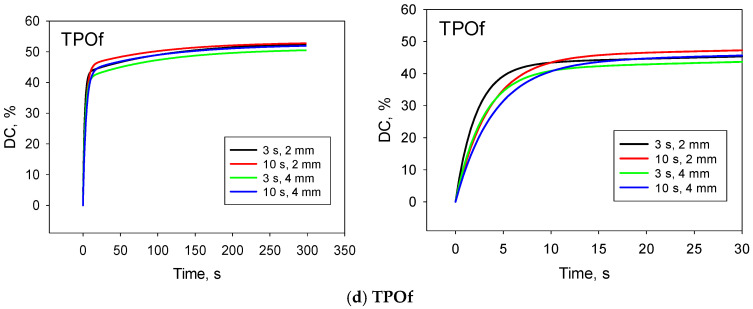
Temporal DC evolution from the beginning of polymerization to 300 s after polymerization (left) with a close-up of the first 30 s (right) as a function of curing mode and depth for analysed materials (**a**) TPL; (**b**) TPLf; (**c**) TPO; (**d**) TPOf (abbreviations in Table 1).

The kinetics of polymerization are shown for each material as a function of curing mode and depth over the observation period of 300 s, along with a close-up of the first 30 s to evidence differences in the rate of DC increase under different curing conditions (Figure 4).

The parameters of the exponential sum functions describing the temporal DC variation for all materials, curing conditions, and material depths are summarised in Table 2. With R^2^ values greater than 0.93 in all groups, the fit of the exponential used model is very good. Furthermore, parameters a and b characterise the gel phase of the polymerization process, and parameters d and c characterise the glass phase.

## 4. Discussion

The trend toward simplifying composite application continues with the development of materials that can not only be applied in bulk, in 4 mm increments, and cure ultra-fast [10], but are now also aesthetically pleasing enough to be used in both the posterior and anterior regions. Since these materials have only recently been introduced to the market, there is currently little information available, but their mechanical behaviour has already been extensively analysed [22]. The new composites demonstrate good mechanical properties that are well within the range of clinically successful nano- and micro-hybrid composites [22]. A direct comparison with predecessor materials (TPOf) from the same company shows that the new development in the low-viscosity (flowable) version (TPLf) exhibits higher flexural strength and mechanical work, as well as statistically similar elastic modulus and fracture toughness values. In the high-viscosity formulation, the new composite (TPL) exhibits similar flexural strength and fracture toughness, higher mechanical work, and a slightly lower elastic modulus compared to the previous formulation (TPO) [22].

The analysed materials share the implementation of the reversible addition–fragmentation chain-transfer (RAFT)-mediated polymerization. The change from conventional radical polymerization of methacrylates, commonly used in dental composites, to RAFT-mediated polymerization enabled the breakthrough to a shortened light exposure, with no compromises in curing quality [14]. The technology, which was developed at the end of the last century [18], was obviously difficult to incorporate into dental materials, as it is currently only used in a few bulk-fill composites. Finding the suitable RAFT agent [18] seems to have been the main impediment for the further dissemination of the technology. A specially designed RAFT agent, a β-allyl sulfone [20], has been custom-synthesised and is not commercially available.

In its simplest form, RAFT-mediated polymerization is a conventional radical polymerization carried out in the presence of a RAFT agent [18]. This essentially introduces two additional steps into the polymerization process. Initiation and propagation are similar in both mechanisms. In RAFT-mediated polymerization, a pre-equilibrium step follows, in which the propagating radical reacts with the RAFT agent to form an intermediate radical (RAFT adduct radical). The latter can then undergo a fragmentation reaction in which either the reactants are returned or a radical with an initiating leaving group is released, simultaneously forming a polymer compound that represents the resting species. The subsequent re-initiation applies only to RAFT-mediated polymerization, where the leaving group radical reacts with another monomer to initiate a new active polymer chain. It transits to the main RAFT equilibrium, in which the propagating macroradical reacts again with the polymeric RAFT agent. Termination occurs when active chains react by bi-radical termination, resulting in chains that cannot react further (dead polymer) [18].

For the user, the handling of composites with RAFT-mediated polymerization does not differ significantly from conventional radical polymerization. The technology offers the great advantage of retaining all chemical components as well as the curing units while offering a polymer with a narrow chain length distribution, a defined molecular weight, and a more complex architecture. In contrast, very long chains with a broad chain-length distribution are formed in a radical polymerization as a consequence of the fact that the chains are continuously initiated throughout the polymerization and also frequently terminated. The rate of chain initiation and the rate of chain termination are in equilibrium, resulting in an approximately constant but low concentration of propagating radicals [18].

Although a significantly higher DC is observed for the flowable materials than for the highly viscous materials, this should not be considered a quality criterion, as the chemical composition of the monomer system is different for all four materials, and the DC value can therefore describe different aspects. A comparison of the DC is only possible within an identical monomer system, thus only the effect of the depth and type of light exposure within one material can be assessed. The degree of conversion (DC) determined 300 s after polymerization within a material varies less, depending on the curing depth and curing mode, allowing the statement of a sufficient polymerization under the tested conditions. It should be noted that the two curing conditions differ not only in exposure time (3 s vs. 10 s) and radiant emittance but also in radiant exposure (= product of exposure time and radiant emittance). This, in spite of the very high radiant emittance in the 3sCure mode, is significantly higher in the 10 s High Power mode. The comparison is therefore not entirely balanced and is made to the detriment of the 3sCure mode, as the total radiant exposure is about 80% lower here. Furthermore, the radiant exposure in the violet wavelength range in High Power mode is more than twice that of 3sCure, which may have a positive effect on the initiation of the Norrish type I photoinitiators used in these materials, namely Ivocerin and the acylphosphine oxide [23]. Ivocerin, a dibenzoylgermanium derivative (bis-(4-methoxybenzoyl)diethylgermane), which was described by Moszner et al. [24] as an initiator with a lower absorption maximum (λ_max_ = 411 nm and λ_max_ = 418 nm for two different variants) compared to camphorquinone (CQ, λ_max_ = 468 nm), matches well with the emission spectra of the used LCU, which show a maximum peak at 409 nm in the violet wavelength range. Since the absorption spectra of Ivocerin extend up to 460 nm, it also benefits from the blue wavelength range of the LCU. Furthermore, Ivocerin was found to exhibit higher reactivity compared to CQ, a fact attributed to the higher molar extinction coefficient (ελ) [24], which makes it a more efficient initiator. This partially compensates for the disadvantages that might arise from the overall lower RE in the 3sCure mode and may explain the quite similar DC values measured at 300 s post-polymerization. Differences are only visible in the polymerization kinetics, which is well described by the sum of two exponential functions, the first describing the gel phase and the second the glass phase. The first exponential function, described by parameters a and b, indicates a lower parameter b within one material in the 10 s High Power curing mode compared to the 3sCure, thus expressing a slower transition rate of the aliphatic C=C functional groups of the monomers to C-C groups upon incorporation into the polymer. A similar situation applies to increasing depth, as light is scattered and decreases exponentially with the depth of penetration into the material, so that a lower amount of light reaches a depth of 4 mm than a depth of 2 mm. However, this small difference is offset by the glass phase, which is described by parameters c and d, where parameter c is higher in the DC measured at 4 mm than at 2 mm, thus compensating for the initially slower rate of DC rise.

Based on the investigations conducted in this study, the null hypothesis that (a) the DC at 300 s post-polymerisation is not affected by the curing mode (3sCure for 3 s vs. High Power for 10 s) and depth (2 mm vs. 4 mm) is accepted, while hypothesis (b) that the polymerisation kinetics are similar is rejected. Taking into account the differences in the chemical composition of the 4 materials and consequently the fact that DC cannot be directly compared, it can still be stated that the new materials behave similarly to their predecessors in terms of polymerization behaviour and are suitable to be cured in 3 s with high irradiance in 4 mm thick increments. The profile of their mechanical performance, with the limitation of the scarce information available so far as described above, allows the expectation of comparable clinical behaviour, but this needs to be demonstrated in clinical studies.

## 5. Conclusions

Novel bulk-fill composites with RAFT-mediated polymerization demonstrate degree of conversion results that were independent of the measured sample depth or curing mode. They are therefore suitable for rapid curing (3 s) with high irradiance. The polymerization kinetics are somewhat slower in the gel phase at moderate irradiance or when measured at deeper levels but equalise in the glass phase, reaching equivalent conversion levels by the end of the 300-s observation period.

## Figures and Tables

**Table 1:** Analysed composites: brand, abbreviation (code), shade, batch, monomer composition, and filler quantity (weight and volume percent) as provided by the manufacturer (Ivoclar Vivadent, Schaan, Liechtenstein).

Composite	Code	Shade	LOT	Monomer	Filler wt%/vol%
Tetric^®^ PowerFill	TPO	^IV^A	Z04FD5	Bis-GMA, Bis-PMA, UDMA, Bis-EMA, TDM	76–77/53–54
Tetric^®^ PowerFlow	TPOf	^IV^A	Z05FP4	Bis-EMA, Bis-GMA, TDM	68.2/46.4
Tetric^®^ plus Fill	TPL	A3 plus	YM0202	UDMA; AAUDMA; TDM	70/51–52
Tetric^®^ plus Flow	TPLf	A3 plus	YM0210	2-(2-Phenylphenoxy)ethyl 2-methylprop-2-Enoat TDM, UDMA N-(2-Methacryloyloxyethyl)carbaminsäure-(2-methacryloyloxyethyl)ester AAUDMA	65/50–51

Abbreviations: Bis-GMA: bisphenol A-glycidyl methacrylate; TEGDMA: triethylene glycol dimethacrylate; UDMA: urethane dimethacrylate; Bis-EMA: ethoxylated bisphenol A dimethacrylate; TDM: tricyclodocane dimethanol dimethacrylate; AAUDMA: aromatic aliphatic urethane dimethacrylate; wt: weight; vol: volume.

**Table 2:** Parameters of the exponential sum functions describing the polymerization kinetics in analysed materials, curing conditions, and sample depths. The lower (min.) and upper (max.) values of the 95% confidence intervals of these parameters are given.

Composite	Depth	Exposure	R^2^	a_min_	a_max_	b_min_	b_max_	c_min_	c_max_	d_min_	d_max_
TPL	2 mm	3 s	0.95	36.02	36.41	0.28	0.29	6.63	6.94	0.01	0.01
		10 s	0.95	35.12	35.59	0.27	0.28	7.37	7.76	0.01	0.01
	4 mm	3 s	0.95	32.74	33.22	0.23	0.25	8.26	8.64	0.01	0.01
		10 s	0.95	34.70	35.29	0.15	0.16	7.70	8.13	0.01	0.01
TPLf	2 mm	3 s	0.98	45.50	45.76	0.47	0.48	8.28	8.50	0.01	0.01
		10 s	0.98	47.49	47.77	0.33	0.34	7.70	7.93	0.01	0.01
	4 mm	3 s	0.99	44.07	44.35	0.29	0.30	9.30	9.54	0.01	0.01
		10 s	0.98	42.95	43.22	0.28	0.29	9.67	9.91	0.01	0.01
TPOf	2 mm	3 s	0.98	42.87	43.10	0.46	0.47	10.15	10.36	0.01	0.01
		10 s	0.98	45.13	45.38	0.28	0.29	7.84	8.05	0.01	0.01
	4 mm	3 s	0.99	41.08	41.31	0.35	0.35	9.74	9.93	0.01	0.01
		10 s	0.99	43.34	43.62	0.25	0.25	8.88	9.10	0.01	0.01
TPO	2 mm	3 s	0.93	36.15	36.61	0.41	0.43	8.11	8.50	0.01	0.01
		10 s	0.95	38.05	38.48	0.28	0.29	7.29	7.64	0.01	0.01
	4 mm	3 s	0.93	32.72	33.24	0.30	0.31	7.47	7.91	0.01	0.01
		10 s	0.95	35.04	35.57	0.21	0.22	8.26	8.67	0.01	0.01

## Data Availability

The original contributions presented in this study are included in the article. Further inquiries can be directed to the corresponding author.

## References

[1] Sarapultseva M., Hu D., Sarapultsev A. (2025). Clinical Performance of Bulk-Fill Versus Incremental Composite Restorations in Primary Teeth: A Systematic Review of In Vivo Evidence. Dent. J..

[2] Hofmann M., Wolf E., Lücker S., Frankenberger R., Wöstmann B., Krämer N. (2025). Marginal Quality and Wear of Bulk-Fill Composites: Differences Between Dentitions. J. Adhes. Dent..

[3] Lucchi P., Mazzoleni S., Parcianello R.G., Gatto R., Gracco A., Stellini E., Ludovichetti F.S. (2024). Bulk-flow composites in paediatric dentistry: Long term survival of posterior restorations. A retrospective study. J. Clin. Pediatr. Dent..

[4] de Menezes A.J.O., do Nascimento Barbosa L., Leite J.V.C., Barbosa L.M.M., Montenegro R.V., Dantas R.V.F., de Souza G.M., de Andrade A.K.M., Lima R.B.W. (2025). Clinical Outcomes of Bulk-Fill Resin Composite Restorations: A 10-Year Mapping Review and Evidence Gap Map. J. Esthet. Restor. Dent..

[5] Schoilew K., Fazeli S., Felten A., Sekundo C., Wolff D., Frese C. (2023). Clinical evaluation of bulk-fill and universal nanocomposites in class II cavities: Five-year results of a randomized clinical split-mouth trial. J. Dent..

[6] Rueggeberg F.A. (2011). State-of-the-art: Dental photocuring—A review. Dent. Mater..

[7] Deb S., Sehmi H. (2003). A comparative study of the properties of dental resin composites polymerized with plasma and halogen light. Dent. Mater..

[8] Randolph L.D., Palin W.M., Watts D.C., Genet M., Devaux J., Leloup G., Leprince J.G. (2014). The effect of ultra-fast photopolymerisation of experimental composites on shrinkage stress, network formation and pulpal temperature rise. Dent. Mater..

[9] Joly G.D., Abuelyaman A.S., Fornof A.R., Craig B.D., Krepski L.R., Moser W.H., Yurt S., Oxman J.D., Falsafi A. (2015). Dental Compositions Comprising Addition-Fragmentation Agents (Patent). https://patents.google.com/patent/US9907733B2/en.

[10] Ilie N., Watts D.C. (2020). Outcomes of ultra-fast (3 s) photo-cure in a RAFT-modified resin-composite. Dent. Mater..

[11] Watts D.C., Algamaiah H. (2020). Characterizing surface viscoelastic integrity of ultra-fast photo-polymerized composites: Methods development. Dent. Mater..

[12] Par M., Marovic D., Attin T., Tarle Z., Tauböck T.T. (2020). Effect of rapid high-intensity light-curing on polymerization shrinkage properties of conventional and bulk-fill composites. J. Dent..

[13] Marovic D., Par M., Daničić P., Marošević A., Bojo G., Alerić M., Antić S., Puljić K., Badovinac A., Shortall A.C. (2025). The Role of Rapid Curing on the Interrelationship Between Temperature Rise, Light Transmission, and Polymerisation Kinetics of Bulk-Fill Composites. Int. J. Mol. Sci..

[14] Ilie N., Diegelmann J. (2021). Impact of ultra-fast (3 s) light-cure on cell toxicity and viscoelastic behavior in a dental resin-based composite with RAFT-mediated polymerization. J. Mech. Behav. Biomed. Mater..

[15] Baabdullah F., Bakitian F., Alshammari H., Aljanakh M., Holdar A., Ozcan I., Antonson S.A. (2025). Comparative analysis of pulpal temperature changes in bulk fill resin composites with different photoinitiators activated using various light curing units: An in vitro study. Dent. Mater..

[16] Klarić E., Bosnić J.V., Par M., Tarle Z., Marovic D. (2024). One-Year Evaluation of High-Power Rapid Curing on Dentin Bond Strength. Materials.

[17] Par M., Spanovic N., Marovic D., Attin T., Tarle Z., Tauböck T.T. (2021). Rapid high-intensity light-curing of bulk-fill composites: A quantitative analysis of marginal integrity. J. Dent..

[18] Beres M.A., Boyer C., Hartlieb M., Konkolewicz D., Qiao G.G., Sumerlin B.S., Perrier S. (2025). RAFT with Light: A User Guide to Using Thiocarbonylthio Compounds in Photopolymerizations. ACS Polym. Au.

[19] Barner-Kowollik C. (2008). Handbook of RAFT Polymerization.

[20] Gorsche C., Griesser M., Gescheidt G., Moszner N., Liska R. (2014). β-Allyl Sulfones as Addition–Fragmentation Chain Transfer Reagents: A Tool for Adjusting Thermal and Mechanical Properties of Dimethacrylate Networks. Macromolecules.

[21] Ilie N. (2023). An In Vitro Comparison of Elastoplastic and Viscoelastic Behavior of Dental Composites with Reversible Addition–Fragmentation Chain Transfer-Mediated Polymerization. J. Compos. Sci..

[22] Ilie N. (2025). Relationship Between Fracture Toughness and Fracture Mirror in Modern Polymer-Based Dental Composites. J. Funct. Biomater..

[23] Fernández Godoy E., Chaple Gil A., Caviedes Thomas R., Bersezio Miranda C., Martín Casielles J., Rodríguez Martínez G., Angel Aguirre P. (2025). Efficiency and limitations of polywave light-curing units in restorative dentistry: A systematic review. Clin. Oral. Investig..

[24] Moszner N., Fischer U.K., Ganster B., Liska R., Rheinberger V. (2008). Benzoyl germanium derivatives as novel visible light photoinitiators for dental materials. Dent. Mater..

